# Dysentery

**Published:** 1828-07

**Authors:** 


### DYSENTERY.

*Cases of Dysentery, in which the Hydrochloruret of Lime was employed, by* Dr. Reid, *at the* Fever and Dysentery Hospital, Dublin.

I.—John Coyne was admitted into the hospital in the last stage of dysentery, which came on after a tedious fever. The discharges consisted of a bloody sanies, intermixed with a dark-coloured matter, the smell of which was quite overpowering and offensive. The stools were passed involuntarily, and the patient was reduced to an idiotic state. I considered that erosion of the intestines had taken place, so that his death was inevitable. I directed ten grains of the Hydrochloruret of Lime to be added to the common enema of the Pharmacopœia, and to be administered to the patient night and morning. The stench, which was so intolerable, was soon corrected, the evacuations became more natural in appearance, and pure blood was sometimes discharged. His tongue became clean, and grew moist, but exhaustion continued to increase; notwithstanding which, he survived a fortnight after his admission.

II.—I directed similar injections for Rose Macnamara, who was labouring under dysentery after fever, in the female convalescent ward. On the 11th of November, 1826, the stench from the incessant discharges from her bowels was so intolerable, that the other patients declared they could not continue in the ward. The pain she suffered was at times excruciating.

The first day after the injections with the hydrochloruret were employed, the patient experienced considerable relief of all the symptoms. In a few days the fœtor was corrected. The progress of the cure was gradual and steady, and she was discharged well on the 6th December. She has continued perfectly well to the present moment.

III.—James Heron, a very old man, and apparently of a broken down constitution, was admitted on the 22d March, affected with dysentery. He had frequent evacuations, occasionally mixed with blood, and attended with great pain in the abdomen. Tongue extremely loaded; urgent thirst; great debility; some appetite. He became convalescent from a tedious and severe fever on February 20th. The dysenteric symptoms came on soon afterwards, and progressively increased up to the present time. I directed a mixture with Acetate of Ammonia and Camphorated Tincture of Opium; but, not finding any relief from this treatment, I directed on the 24th a mixture, consisting of ten grains of the Hydrochloruret of Lime, with two drachms of the Tincture of Columbo, in four ounces of water and two drachms of syrup; half an ounce to be taken every hour.

25th.—His tongue began to get moist, though still loaded with brown fur.—Perstet.

26th.—Tongue cleansing; evacuations changing to a more natural appearance. He has not had occasion to go to the chair more than eight or ten times in the twenty-four hours. Longer intervals from pain, but at times he thinks it more severe than before.—Perstet.

27th.—Feels better in all respects; tongue cleansing.

**Let him take his mixture only every third hour.**

28th.—No pain since last visit. Was up but four times. Evacuations nearly natural; says he feels himself almost well.

**Let him take his mixture only night and morning.**

29th.—Convalescent.

31st.—Discharged cured, and says he has not felt himself so well in health for many years.

IV.—William Carl, admitted on 31st March, affected with dysentery upwards of three weeks. Complains of severe pain in his bowels; incessant alvine evacuations of thin fœtid matter, intermixed with a quantity of bloody sanies; great emaciation and debility; some appetite. No rest at night, owing to the constant necessity of relieving his bowels from their morbid contents. Some swelling of the abdomen, and his legs œdematous. Had been upwards of six weeks in Sir Patrick Dun's Hospital, under treatment for dropsy. Had been tapped once while in that hospital, and a vast quantity of water, he says, was drawn off. His present disease commenced about a week after having been discharged from Sir Patrick Dun's.

On the 2d April commenced regular treatment with Hydrochloruret of Lime. During the first two days from his admission, I considered it necessary to give him medicine, so as to bring him into a proper state to employ this substance with effect. I now directed ten grains of the Hydrochloruret of Lime to be dissolved in four ounces of diluted syrup, to which were added twenty drops of essential Oil of Carraway; half an ounce to be taken every hour.

5th April.—Continued the medicine as directed on the 2d; feels in general rather better; some evacuations from his bowels, now not tinged with blood.

7th.—No blood since yesterday; pains in abdomen very slight, and seldom.—Perstet.

9th.—Still mending in all respects; swelling of limbs subsided.

Let him take his medicine only three times a day.

11th.—Mending in every respect. Countenance filling up. Stools now slimy, without pain or uneasiness.

Let him take the mixture only night and morning.

12th.—At stool but six times last twenty-four hours. Evacuations thin and whitish. No pain. No blood passed. Countenance much improved. Strength returning. Tongue moist with white mucous covering. He has now his clothes on, and is able to walk.*

---

